# TNFAIP8/TIPE2 inactivation modulates extracellular matrix organization gene expression and preserves the intervertebral disc structure in mice

**DOI:** 10.1016/j.gendis.2025.101835

**Published:** 2025-08-30

**Authors:** Zuozhen Tian, Ken Chen, Frances S. Shofer, Srish S. Chenna, Daniel Z. Sandroni, Ling Qin, Yejia Zhang

**Affiliations:** a Department of Physical Medicine & Rehabilitation, Children's Hospital of Philadelphia, Philadelphia, PA 19146, USA; b Department of Orthopedics, Xiangya Hospital, Central South University, Changsha, Hunan 410008, China; c Department of Emergency Medicine, Perelman School of Medicine, University of Pennsylvania, Philadelphia, PA 19104, USA; d Section of Rehabilitation Medicine, Corporal Michael J. Crescenz Veterans Affairs Medical Center, University of Pennsylvania, Philadelphia, PA 19104, USA; e Department of Orthopedic Surgery, Perelman School of Medicine, University of Pennsylvania, Philadelphia, PA 19104, USA; f School of Dental Medicine, University of Pennsylvania, Philadelphia, PA 19104, USA

Back pain related to intervertebral disc (IVD) degeneration is a common clinical problem. Inflammatory cytokines and chemokines have been found in painful/degenerative human IVDs^1^ and may account for some painful symptoms. The TNFAIP8 (tumor necrosis factor-α-induced protein 8) family, discovered by Chen and colleagues via genomic profiling of inflamed tissues, comprises four highly homologous mammalian proteins, designated TNFAIP8 and TIPE1-3 (TNFAIP8-like 1-3, or TIPE1-3). TNFAIP8 and TIPE2 direct leukocyte migration and fine-tune inflammation.^2^^,^^3^ *Tnfaip8* and *Tipe2* gene expression is perturbed by injury to mouse IVDs,^4^ suggesting that the products of these genes play a role in injury/repair responses. Furthermore, TNFAIP8 and TIPE2 loss of function ameliorated immediate proteoglycan loss and inflammation in the injured IVDs.^5^ Here, we examined the effects of their function loss on the IVDs in mice in which both genes were deleted. Transcriptome and morphological features of IVDs in male mice 10–12 weeks of age were analyzed. *Tnfaip8/Tipe2* double knockout (dKO) mice were compared with wild type (WT) mice on the same genetic background. Four consecutive intact coccygeal IVDs (C3/4. C4/5, C5/6, and C6/7) were pooled for RNA extraction.

IVDs in *Tnfaip8/Tipe2*-dKO and WT mice displayed different gene expression profiles***.*** A list of differentially expressed genes was generated based on 17 996 genes with meaningful readings via RNA-seq. A total of 349 genes differed between dKO and WT (Wald test; *P.adj* < 0.01). The genes were further sorted according to a fold change ≥2, revealing 44 up-regulated and 22 down-regulated genes in the *Tnfaip8/Tipe2*-dKO mice compared with the WT controls. Among these genes, those with the highest fold changes comparing WT with dKO mice were selected for heatmap, which clearly reveals different expression profiles between WT and mutant mice (Fig. 1A).

**Figure 1:**
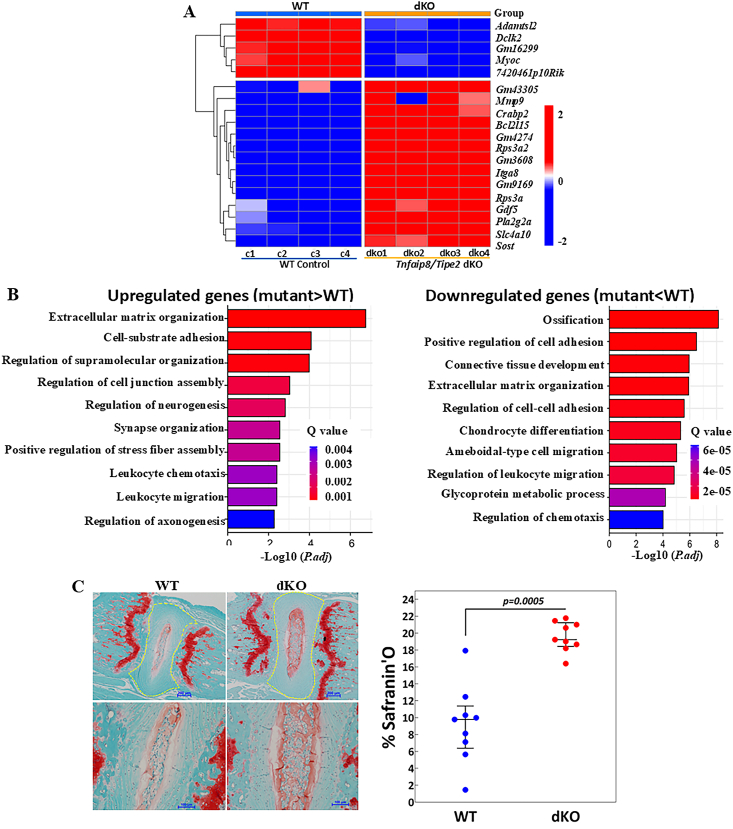
Gene expression and morphological features of the intervertebral discs of wild type (WT) and *Tnfaip8/Tipe2* double knock out (dKO) mouse. **(A)** Heatmap of genes with the highest fold changes; *Adamtsl2:* ADAMTS like 2; *Dclk2*: Doublecortin Like Kinase 2; *Myoc:* myocilin; *7420461p10Rik:* myocilin opposite strand (Myocos); *Mmp9:* Matrix metalloproteinase-9; *Crabp2:* cellular retinoic acid binding protein 2; *Bcl2l15:* BCL2 like 15; *Rps3a:* ribosomal protein S3A; *Itga8:* Integrin Subunit Alpha 8; *Gdf5:* growth differentiation factor 5; *Pla2g2a:* Phospholipase A2 Group IIA; *Slc4a10:* Solute Carrier Family 4 Member 10; *Sost:* Sclerostin. **(B)** Pathway analysis; *P.adj*: adjusted *P*-value. Q value: estimated false discovery rate; **(C)** Histological features; Yellow outlined areas on the left panel were quantified for % Safranin'O staining, shown on the right panel; Each symbol represents one mouse on the right panel; *n* = 9 mice/group; error bar: median ± interquartile range.

Gene Ontology (GO) analysis for biological processes was performed to give meaning to the 349 differentially expressed genes. Extracellular matrix organization and cell-substrate adhesion were the two most represented biological pathways among the up-regulated genes, while ossification was the most represented pathway among the down-regulated genes (Fig. 1B). Protein–protein interaction network analysis revealed that fibronectin 1 (FN1) and thrombospondin 1 (THBS1) had the highest number of protein–protein interactions (Fig. S1). It is worth noting that the data presented are based on RNA-seq, and confirmatory real-time PCR for selected genes is an important future direction.

The *Tnfaip8/Tipe2*-dKO mouse IVDs displayed higher levels of Safranin'O staining compared with WT. In permanently mounted sections, Safranin'O seems to bind only to tissue polyanions and not to collagen, and is therefore used as a quantitative histochemical marker for the concentration of mucopolysaccharides in IVDs. The intact nucleus pulposus (NP) was stained red in both the dKO and WT mouse discs (Fig. 1C, left panels). Quantification by ImageJ (NIH Imaging) revealed that *Tnfaip8/Tipe2*-dKO mouse IVDs stained more intensely for Safranin'O than did WT mice (Wilcoxon rank sum test; median = 19.2 *vs* 9.7, *P* = 0.0005; Fig. 1C, right panel).

Normal to a very mild level of degeneration was observed in WT discs, as assessed using histological scoring based on microscopic sections stained with hematoxylin and eosin. The scoring process involved 2–3 graders, and their evaluations showed good correlation, indicating consistent results (*n* = 28 mice; intraclass correlation coefficients (ICC) = 0.93). The average histological scores for the intact IVDs of both WT and dKO mice fell within the normal range (0–6). However, the scores of WT mouse IVDs were significantly higher than those of dKO mice (median = 2 and 0; *P* = 0.0010; Fig. S1). The main histological differences between the IVDs of the WT and dKO mice were in the NP area (Table S1).

Note that data on tail IVDs have been shown here, and comparing changes in lumbar spine histological features would be an important future direction. Another limitation is that only male mice were used in this study. In the future, it is important to compare male and female mice since sex affects gene expression and tissue structure.

In summary, an unbiased bulk RNA-seq approach was used to compare the transcriptomes of *Tnfaip8/Tipe2*-dKO and WT control mouse IVDs. Significant differences in the gene expression profiles were detected between the mutant and WT mouse IVDs. Importantly, *Tnfaip8/Tipe2*-dKO mouse discs retained more proteoglycans than WT mice. Although the TNFAIP8 family play important roles in regulating immune function and are likely required for survival, reduced expression locally in the disc during development may be beneficial in preventing tissue degeneration.

## CRediT authorship contribution statement

**Zuozhen Tian:** Methodology, Data curation. **Ken Chen:** Visualization, Software, Methodology. **Frances S. Shofer:** Visualization, Methodology, Formal analysis. **Srish S. Chenna:** Methodology, Data curation. **Daniel Z. Sandroni:** Visualization, Methodology, Data curation. **Ling Qin:** Project administration, Methodology, Conceptualization. **Yejia Zhang:** Writing – review & editing, Writing – original draft, Visualization, Project administration, Methodology, Funding acquisition, Formal analysis, Data curation, Conceptualization.

## Ethical approval

All animal use in this study was reviewed and approved by the Institutional Animal Care and Use Committee (IACUC) at the University of Pennsylvania, Philadelphia, PA. All animal experimental procedures were carried out in compliance with the Animal Research: Reporting of In Vivo Experiments (ARRIVE) guidelines.

## Data availability

The RNA-seq datasets generated and analyzed during this study will be available upon reasonable request to the corresponding author.

## Funding

This work was supported, in part, by research grants to YZ from the Department of Veterans Affairs Healthcare Network and a grant from the 10.13039/100000069National Institute of Arthritis and Musculoskeletal and Skin Diseases (NIAMS, R21 AR071623). The histology core facility has been supported by a grant to the 10.13039/100016787Penn Center for Musculoskeletal Disorders (PCMD; P30AR069619).

## Conflict of interests

The authors have no financial or other conflict of interests. None of the authors have any professional or financial affiliations that may be perceived to have biased the presentation.
