# Supplementary material for: TNFAIP8/TIPE2 inactivation modulates extracellular matrix organization gene expression and preserves the intervertebral disc structure in mice
Source: Genes Dis. 2025 Aug 30;13(3):101835. doi: 10.1016/j.gendis.2025.101835 (PMC12828491; doi:10.1016/j.gendis.2025.101835)
Supplement: Multimedia component 1 [file mmc1.docx]

**Supplementary Materials and Methods.**

***Mice, intervertebral disc tissue isolation and RNA extraction.*** A breeding pair of *Tnfaip8/Tipe2*-double knockout (dKO) mice was gifted by Dr. Youhai Chen (Perelman School of Medicine at the University of Pennsylvania). C57BL6 mice (the Jackson Laboratory, Bar Harbor, ME, USA) were bred as WT controls. Mice were housed in the same facility, under pathogen-free conditions with environmental enrichment. A total of 36 male mice (17 *Tnfaip8/Tipe2*-dKO and 19 WT mice), age 10-12 weeks, were used. Among these, 8 mice were used for RNASeq, and 28 mice for histological studies.

***RNASeq.*** A total of 8 male mice (4 *Tnfaip8/Tipe2*-dKO and 4 WT mice) were used for RNASeq. Four consecutive intact coccygeal IVDs (C3/4. C4/5, C5/6, and C 6/7) were pooled for RNA extraction. Specifically, IVDs were isolated under a dissection microscope, soaked in RNALater, and stored in a -80 ^0^C freezer. Total cellular RNA was extracted by the Trizol method, and purified using a RNeasy Micro Kit (Qiagen). RNA Library Preparation and Sequencing was performed by Azenta US (South Plainfield, NJ). Specifically, RNA samples were quantified using Qubit 2.0 Fluorometer (ThermoFisher Scientific, Waltham, MA, USA) and RNA integrity was checked with 4200 TapeStation (Agilent Technologies, Palo Alto, CA, USA). rRNA depletion sequencing library was prepared by using QIAGEN FastSelect rRNA HMR Kit (Qiagen, Hilden, Germany). RNA sequencing library preparation uses NEBNext Ultra II RNA Library Preparation Kit for Illumina by following the manufacturer’s recommendations (NEB, Ipswich, MA, USA). Briefly, enriched RNAs were fragmented for 15 minutes at 94°C. First strand and second strand cDNA were subsequently synthesized. cDNA fragments were end-repaired and adenylated at 3’ends, and universal adapters were ligated to cDNA fragments, followed by index addition and library enrichment with limited cycle PCR. Sequencing libraries were validated using the Agilent Tapestation 4200 (Agilent Technologies, Palo Alto, CA, USA), and quantified using Qubit 2.0 Fluorometer (ThermoFisher Scientific, Waltham, MA, USA) as well as by quantitative PCR (KAPA Biosystems, Wilmington, MA, USA). The sequencing libraries were multiplexed and clustered on one flowcell. After clustering, the flowcell was loaded on the Illumina HiSeq instrument according to the manufacturer’s instructions. The samples were sequenced using a 2x150 Pair-End (PE) configuration Raw sequence data (.bcl files) generated from Illumina HiSeq were converted into fastq files and de-multiplexed using Illumina bcl2fastq program version 2.20. One mismatch was allowed for index sequence identification.

***RNASeq data Analysis****.* After demultiplexing, sequence data were checked for overall quality and yield. Then, sequence reads were trimmed to remove possible adapter sequences and nucleotides with poor quality using Trimmomatic v.0.36. The trimmed reads were mapped to the reference genomes using the STAR aligner v.2.5.2b. The STAR aligner is a splice aware aligner that detects splice junctions and incorporates them to help align the entire read sequences. BAM files were generated as a result of this step. Unique gene hit counts were calculated by using feature counts from the Subread package v.1.5.2. Only unique reads within exon regions were counted. After extraction of gene hit counts, the gene hit counts table was used for downstream differential expression analysis. Using DESeq2, a comparison of gene expression between the groups of samples was performed. The Wald test was used to generate p-values and Log2 fold changes.

***Generation of Heatmap.*** 17,996 genes examined by RNASeq had meaningful readings. A differential expressed gene list was generated by limiting *P.adj* to <0.01, resulting in 349 genes in this category. The genes were further sorted according to log2 fold changes from high to low, resulting in 44 upregulated and 22 downregulated genes in the *Tnfaip8/Tipe2*-dKO mice compared with WT controls. For upregulated genes, we selected 14 genes based on their highest rankings. For downregulated genes, we selected the 5 genes with highest fold changes comparing WT with *Tnfaip8/Tipe2*-dKO mice. After inputting the chosen upregulated genes and downregulated genes, a heatmap was generated using R package pheatmap, in which rows (which represent gene expression) were scaled and hierarchically clustered, and a gap was inserted among columns to differentiate the WT and dKO groups.

**Gene ontology (GO).** A protein list was generated based on fold-changes. GO analysis of the 349 genes with *p.adj*<0.01 was performed for biological processes with the R software.

**Protein-Protein Interaction (PPI) Network Visualization**. Ranking PPI networks were downloaded from STRING database (https://string-db.org/, Version: 11.5) and imported into CytoScape software (Version: 3.9.1). The minimal interaction score was set at high confidence (0.70). Confidence scores are scaled between 0 and 1, with 1 corresponding to the estimated likelihood of a given association being true. Disconnected nodes in the network were not displayed. CytoHubba plugin was used to rank nodes with the MCC method. The parameters of nodes and edges were adjusted based on node rankings and the combined interaction scores predicted by STRING database, thus visualizing PPI networks in a more intuitive and clear way.

**Histological Preparation.** Twenty-eight male mice, (15 *Tnfaip8/Tipe2*-dKO and 13 WT), age 10-12 weeks were used for histology. Following euthanasia, the coccygeal (C) 5/6 and 6/7 IVDs along with adjacent bony vertebral bodies were promptly isolated. The disc, together with its surrounding vertebral bodies, was fixed in a 4% paraformaldehyde solution for 24 hours. Subsequently, the bone-disc-bone segments underwent decalcification using a 12.5% EDTA solution, with periodic shaking, until complete decalcification of the bony portion was achieved. After decalcification, the tissues were dehydrated, embedded in paraffin, and then sectioned to 4 μm thickness, stained with hematoxylin and eosin (H&E) or Safranin’O.

**Quantification of Safranin’O staining.** Safranin’O, a basic dye, was employed to stain cartilage components (proteoglycans, chondrocytes, and type II collagen), resulting in varying shades of red. The intensity of the Safranin’O staining corresponds directly to proteoglycan levels in the cartilage tissue. Serial section of the tissues described above were stained with Safranin’O. For consistency, only the sections stained at the same time were quantified for staining intensity. To analyze the stained sections, digitalization was performed, and ImageJ software (NIH Image) was utilized. The IVD regions were selectively cropped, and then converted into an HSB Stack. The specific range for the red color was defined with hue values between 220 and 255, and between 0 and 32. Subsequently, the pixel numbers for the red color range and the entire color spectrum (hue values 0-255) were obtained, and the percentage of the red color pixel number in the entire color spectrum was calculated.

**Histological Scoring.** The histological grading was conducted based on a current consensus established by experts in the field of IVD studies.(1) In brief, leading researchers in mouse IVD modeling designed a numeric scale to quantify the extent of degeneration. The scoring criteria encompassed the evaluation of extracellular matrix and cell morphology in the NP and AF, NP-AF-endplate border integrity, and endplate morphology. The total scores ranged from 0 to 35, where 0-6 was normal and 35 was the most severely degenerative. Using H&E-stained sections including discs from mice of both genotypes, three clinician-scientists performed evaluations in a double-blinded manner. Subsequently, the total scores for NP and AF were calculated, and the inter-observer variability was assessed.

**Statistics.** Safranin’O pixel proportion (% red) and histological scores were analyzed using the Wilcoxon rank sum test.

**References**

1. Melgoza IP, Chenna SS, Tessier S, Zhang Y, Tang SY, Ohnishi T, Novais EJ, Kerr GJ, Mohanty S, Tam V, Chan WCW, Zhou CM, Zhang Y, Leung VY, Brice AK, Seguin CA, Chan D, Vo N, Risbud MV, Dahia CL. Development of a standardized histopathology scoring system using machine learning algorithms for intervertebral disc degeneration in the mouse model-an ORS spine section initiative. *JOR Spine.* 2021 July 17; 4(2): e1164. PMCID: PMC8313179.


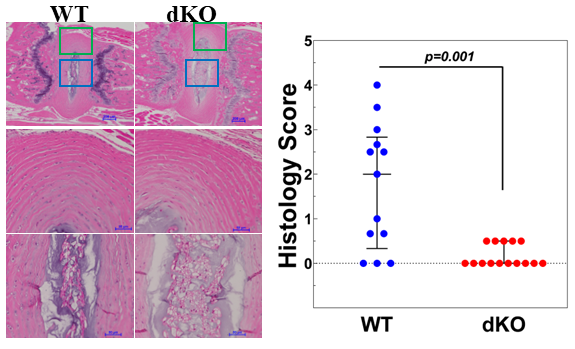


**Figure S1** Histological features of the mouse intervertebral disc (IVD). Left panel: IVD tissues stained with hematoxylin and eosin (HE); C&D and E&F are magnified images of green and blue outlined regions in A&B, respectively. Right panel: Histology score; Each symbol represents one mouse; *n* = 13~~2~~ and 15~~3~~ mice/group; error bar**:** median ± interquartile range.

**Table S1** Histological scoring categories. Scores are presented as mean± standard deviation. NP: nucleus pulposus; AF: annulus fibrosus; dKO: *Tnfaip8/Tipe2* double knockout. Note that only the categories showing signs of degenerative changes are presented in the table. The following categories are not included in the table because they were all scored “0” (no abnormality was found): AF cellularity, lamellar organization, AF clefts/fissures, NP-endplate (EP) boundary, disruption of AF lamella integration to EP, EP cellularity, Fissures & micro-fracture/Schmorl's node.

|  | **NP cellularity** | **NP fibrosis** | **NP matrix organization** | **AF bulging** | **NP-AF boundary** |
| --- | --- | --- | --- | --- | --- |
| **Wild Type** | 0.77±0.60 | 0.38±0.42 | 0.46±0.43 | 0.04±0.14 | 0.08±0.19 |
| **dKO** | 0.17±0.24 | 0 | 0 | 0 | 0 |
| ***p-*value** | 0.004 | 0.006 | 0.002 | 0.337 | 0.165 |
